# Clinical Usefulness of Early Evaluation of the Bacteriological Effect of Antibiotics Administered as Empiric Therapy Using the Fully Automated Urine Particle Analyzer UF‐5000 in Febrile Urinary Tract Infections

**DOI:** 10.1111/iju.70190

**Published:** 2025-08-10

**Authors:** Motohiro Taguchi, Masako Kaido, Naoki Toya, Kumiko Yamada, Akihiro Kanematsu, Toeki Yanagi, Shingo Yamamoto

**Affiliations:** ^1^ Department of Urology Hyogo Medical University Nishinomiya Hyogo Japan; ^2^ Medical & Scientific Affairs Sysmex Corporation Kobe Hyogo Japan; ^3^ Reagent Engineering Sysmex Corporation Kobe Hyogo Japan; ^4^ Department of Clinical Technology Hyogo Medical University Hospital Nishinomiya Hyogo Japan

**Keywords:** antibacterial agents (β‐lactam), bacteriuria monitoring, early detection, fully automated urine particle analyzer UF‐5000, urinary tract infections

## Abstract

**Objectives:**

Antimicrobial susceptibility test results following urine cultures over 3 days indicate that clinical symptoms and overall patient condition should be closely monitored to ascertain the effectiveness of a specific antibiotic course. A Fully Automated Urine Particle Analyzer UF‐5000 (UF‐5000) was used to detect bacteria in the urine. This study evaluated whether clinical urine samples could be analyzed using UF‐5000 for rapid assessment of empiric antibiotic efficacy in treating febrile urinary tract infection.

**Methods:**

The optimal exposure time to antibiotics was estimated to determine the susceptibility of bacterial strains in an in vitro culture system using the UF‐5000. To determine the optimal timing for rapid evaluation of the efficacy of empiric antibiotics in patients with febrile urinary tract infection, the bacterial number and morphological changes in the urine were examined over time using the UF‐5000 and compared with Gram staining results.

**Results:**

In vitro experiments showed that the optimal conditions for monitoring the bacterial count on the BACT scattergram of the UF‐5000 were 6 h after antibiotic administration. In 10 clinical cases, empiric antibiotics generally showed good susceptibility to the dominant pathogen, and the BACT scattergram showed a significant decrease in bacterial counts after 6 h, but bacterial counts increased 24 h after antibiotic administration in three cases; in two cases, the antimicrobial treatment effects were inadequate based on clinical assessment, and in other cases, patients had to undergo multiple treatments.

**Conclusion:**

Monitoring bacterial counts during empiric antibiotic therapy using the UF‐5000 can help predict the effectiveness of antimicrobial treatment.

**Abbreviations and Acronyms**
UTIs: urinary tract infections
AST: antimicrobial susceptibility test
MIC: minimum inhibitory concentration
TAZ/PIPC: tazobactam/piperacillin
CTRX: ceftriaxone
LVFX: Levofloxacin

## 1 Introduction

Retrograde urinary tract infections (UTIs) are caused by uropathogenic *Enterobacteriales* [1, 2]. The diagnosis of UTIs is determined by clinical symptoms, urinalysis, and identification of bacteria by urine culture and antimicrobial susceptibility test (AST) of the causative bacteria. While awaiting AST results over 3 days [3], clinicians must closely monitor blood test data—including peripheral white blood cell count and CRP—as well as clinical symptoms such as fever and the general state of the patient. This monitoring helps assess the effectiveness of empiric antibiotic treatment to determine whether to continue or discontinue its administration [4, 5].

The bacteriological effect of empiric antibiotics on causative bacteria can be determined by monitoring bacterial count changes over time in patients with UTI. However, rapid quantification of these bacteria is not possible, since the primary technique used (i.e., urine culture method) requires more than 18 h to yield results [6]. In addition, the results of urine cultures obtained after antibiotic administration are inaccurate because bacteria are affected by antibiotics and cannot grow normally [4, 7]. Gram staining can determine temporal changes in the number of bacteria with semiquantitative accuracy significantly influenced by the subjectivity of the examiners [7].

The Fully Automated Urine Particle Analyzer UF‐5000 (UF‐5000), an in vitro diagnostic instrument based on flow cytometry, determines clinical parameters in human urine and quantifies bacteria per unit volume by plotting a two‐dimensional scattergram within 2 min [8, 9]. In previous studies, bacterial count measurements using the Fully Automated Urine Particle Analyzer UF‐1000*i*, the previous UF‐5000 model, were shown to be an alternative method for culture methods to determine the susceptibility of bacteria to antibiotics using the in vitro systems [10, 11], suggesting that over time, quantitative bacterial count measurements by UF‐5000 in the urine obtained from patients with UTI can rapidly predict the susceptibility and bacteriological effect of the causative bacteria to empiric antibiotics administered in clinical practice.

In this study, we first monitored the number and morphology of several bacterial strains affected by an in vitro culture system using the UF‐5000 and estimated the optimal exposure time for antibiotics to determine their susceptibility. Additionally, we aimed to determine the optimal timing for rapid evaluation of empiric antibiotic efficacy in clinical cases of febrile UTI. To do this, we used the UF‐5000 to characterize the bacteria in terms of their abundance and morphological features in the urine of patients over time.

## 2 Materials and Methods

### 2.1 Bacterial Strains and Antibiotics

Bacterial strains: *Escherichia coli* : clinical isolates EC156 and EC352 [12], ATCC 35218 and ATCC 25922 [13], *Klebsiella pneumoniae* : clinical isolates KP12 and KP27 [12], ATCC 700603 [14].

Antibiotics: Tazobactam/piperacillin (TAZ/PIPC), which was prepared by mixing TAZ (LKT Laboratories Inc., Minnesota, USA) and PIPC (LKT Laboratories Inc.) (1:4), and ceftriaxone (CTRX, Cayman Chemical Company, Michigan, USA) was used as the antibiotic solution.

### 2.2 UF‐5000 Measurement

Bacterial cultures in the in vitro study and urine samples collected in the clinical study were analyzed using a UF‐5000 (Sysmex Corporation, Kobe, Japan) according to the manufacturer's instructions.

Scattergram analysis: BACT (FLH‐FSC) scattergram reflects bacterial cell size and nucleic acid stainability, plotted as purple dots. The bacterial count per microliter was calculated using a UF‐5000. Cluster locations on the BACT scattergram varied by bacterial type. The CAST (FLL_W‐FLL_A) scattergram represents staining intensity of membrane components and cast matrix. Cells were shown as gray‐dotted clusters, which consisted of normal or small‐sized epithelial cells, budding yeast‐like cells, bacterial chains, and WBC clumps; these clusters likely consisted of larger structures with weak staining intensity.

### 2.3 In vitro Experiments

The bacterial culture solution was prepared as follows: Isolated colony was inoculated into Mueller–Hinton broth medium (Becton, Dickinson and Company, NJ, USA) and cultured at 37°C. The bacterial culture medium was prepared by adding the antibiotics at an intermediate concentration range based on the CLSI‐recommended level [12], with sterile water containing an equivalent amount of antibiotics used as a negative control. After incubation at 37°C for 0, 3, 6, and 24 h, the scattergrams were acquired using UF‐5000. The same samples were observed by Gram staining under a microscope ( × 600 magnification).

### 2.4 Clinical Study

This was a prospective clinical study. Subjects: Patients hospitalized with a diagnosis of febrile UTI at the Hyogo Medical University Hospital between October 2022 and September 2024 agreed to participate in this study and provided written informed consent.

Collected urine samples were stored for a maximum of 12 h at 4°C. Urine samples obtained before antibiotic administration were subjected to urine culture, and those obtained before and 6 and 24 h after antibiotic administration were subjected to UF‐5000 measurement and Gram staining. Simultaneously, changes in UF‐5000 results were compared with clinical outcomes to determine the optimal UF‐5000 parameters for predicting AST results and clinical outcomes. Outcomes were categorized as improvement, failure, or delayed improvement. Improvement was defined as patients achieving a normal body temperature and peripheral WBC counts 72 h after starting antibiotic treatment. Failure referred to cases requiring a change of antibiotics at 72 h due to persistent high body temperature and elevated peripheral WBC counts, indicating inadequate development. Delayed improvement referred to patients who continued the same antibiotics despite having no improvement at 72 h after starting the treatment, but showed improvement within 1 week. Patients who had already received antibiotics prior to treatment in our hospital, those with bacterial counts less than 100/μL (10 × 5/mL) by UF‐5000, those with negative urine culture results, those with no bacteria observed using Gram staining, and those with male genital tract infections such as bacterial prostatitis and epididymitis were excluded from this clinical study.

IRB approval number:

Hyogo Medical University 202 010–030, Sysmex Corporation 2020–216.

### 2.5 Microbiological Procedure

Semiquantitative urine culture was performed by incubating 10 μL of urine on Pore Media Sheep Blood Agar, Pore Media MacConkey Agar, and Pore Media MRSA Isolation Medium II (EIKEN CHEMICAL Co. Ltd., Tokyo, Japan) under aerobic conditions for 18 h, and on BY Chocolate Agar (Becton Dickinson Company Ltd.) under CO_2_ conditions for 18 h. Bacterial species were identified using a MALDI Biotyper (Bruker Japan K. K.) or MicroScan WalkAway 96 Plus (Beckman Coulter Inc., CA, USA). AST was performed using a MicroScan WalkAway 96 Plus with MicroScan panels: Pos Combo 1 J, Neg Combo 2 T, Neg Combo 3 T, Neg MIC 4 J, or a customized Eiken Frozen plate (EIKEN CHEMICAL Co. Ltd.). 10 μL of collected urine was observed by Gram staining under a microscope ( × 1000, oil immersion).

## 3 Results

### 3.1 In Vitro Culture Experiment

**FIGURE 1:**
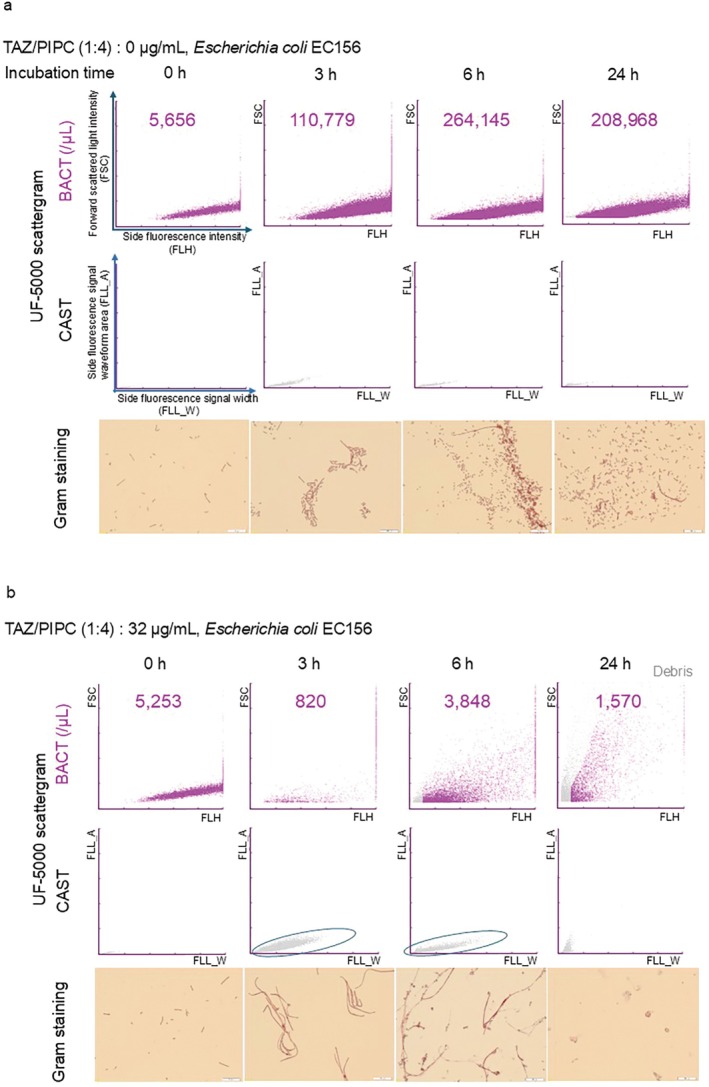
Exemplary detection in UF‐5000 BACT scattergram, CAST scattergram, and Gram staining from in vitro culture experiments. (Upper panels) On the BACT (FLH‐FSC) scattergram of UF‐5000, bacteria were represented by purple dots, and cell fragments were represented by gray dots. The number shown in the scattergram was calculated using UF‐5000 as the count of bacteria per microliter of culture medium. (Middle panels) On the CAST (FLL_W‐FLL_A) scattergram of the UF‐5000, cells appeared in the gray dotted clusters, which included normal or small‐sized epithelial cells, budding yeast‐Glike cells, bacterial chains, and WBC clumps; it was likely to include larger sized structures with weak staining intensity. Circled: Gray dotted cluster on the CAST scattergram for emphasis. (Lower panels) Gram staining; gram‐negative bacteria were stained red. (Figure 1a,b) *Escherichia coli* EC156, a TAZ/PIPC‐susceptible strain.

When *E. coli* EC156 strain (TAZ/PIPC MIC = 0.125 μg/mL) was cultured without TAZ/PIPC, the increase of bacterial number over the course of the culture time was observed using Gram staining, and gram‐negative bacterial clusters appeared on the BACT scattergram of the UF‐5000 (Figure 1a) [15]. When *E. coli* EC156 was cultured in a medium with an intermediate concentration (32 μg/mL), filamentation was observed [16] after 3–6 h, and only aggregated bacterial cells were observed after 24 h with Gram staining. In the UF‐5000 analysis, the bacterial count decreased after 3 h, and clusters specific to gram‐negative bacteria [17] disappeared from the BACT scattergram. Furthermore, purple dots were diffusely plotted on the BACT scattergram after 6 h; thus, the pattern appeared to be different from that without antibiotics. The gray cluster was likely to include a large structure with weak staining intensity on the CAST scattergram, which appeared after 3 and 6 h (Figure 1b, circled). 24 h after antibiotic administration, further agglutination and lysis proceeded, and filamentous bacteria were no longer observed by Gram staining. The BACT scattergram showed more gray dots representing debris, and purple dot clusters diffused, whereas the CAST scattergrams showed cluster disappearance (Figure 1b). When *K. pneumoniae* KP12 (CTRX MIC ≤ 0.06) was cultivated in medium with an intermediate concentration (2 μg/mL), UF‐5000 expressed similar scattergrams to *E. coli* EC156 (Figure S1b). In terms of gram‐positive cocci, TAZ/PIPC‐susceptible *E. faecalis* did not show morphological changes such as filamentation, but showed a recognizable decrease in the bacterial number of UF‐5000 6 h after culturing with TAZ/PIPC (data not shown).

**FIGURE 2:**
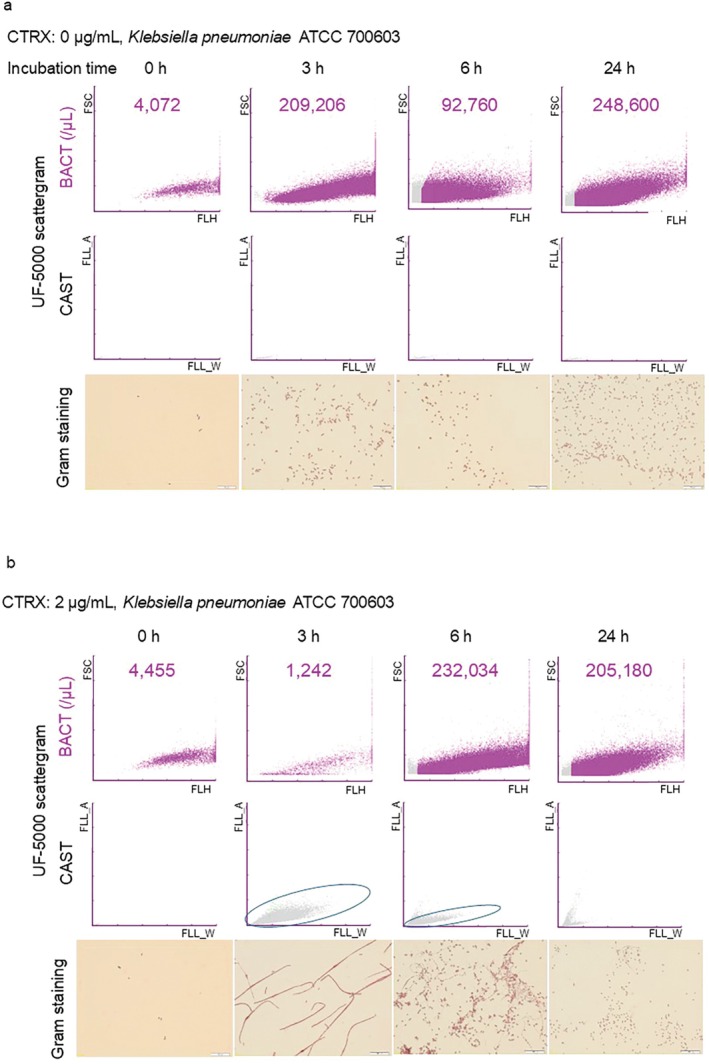
(2a, b) *Klebsiella pneumoniae* ATCC 700603, a CTRX‐resistant strain.

When the highly drug‐resistant strains were incubated at intermediate concentrations, morphological changes were not observed in some strains. For example, *K. pneumoniae* KP27 (CTRX MIC > 128 μg/mL) was detected on the BACT scattergram of UF‐5000 even when incubated with an intermediate concentration (2 μg/mL), showing the same increase in bacterial counts and clustering pattern as when incubated without CTRX, and grew in clusters characteristic of gram‐negative bacteria. No changes were observed in either the CAST scattergram or Gram staining of this strain (Figure S2a,b). However, *K. pneumoniae* ATCC 700603 (CTRX MIC = 16 μg/mL), which MIC is lower than *K. pneumoniae* KP27, was cultured with an intermediate concentration (2 μg/mL); filamentous morphological changes were observed by Gram staining, and clusters representing filamentation appeared on the CAST scattergram simultaneously after 3 h (Figure 2b, circled). The growth of bacteria with normal morphology was observed after 6 h, and most bacteria returned to their normal morphology after 24 h, as noted via Gram staining. BACT scattergrams also showed a decrease in bacterial counts at 3 h after antibiotic addition, whereas a substantial increase in bacterial counts was detected after 6 h; this was in line with the BACT scattergram, which showed an absence of CTRX after 24 h (Figure 2a,b).

When *E. coli* EC352 (TAZ/PIPC MIC = 128 μg/mL) was cultured with an intermediate concentration (32 μg/mL) [12], a similar phenomenon to the culture of *K. pneumoniae* ATCC 700603 with CTRX after 3 h was observed; bacteria began to return to normal clusters after 6 h, and an increase in the number and the normal cluster pattern was observed after 24 h, similar to those observed in the condition without TAZ/PIPC (Figure S3a,b).

### 3.2 Bacterial Changes in Clinical Urine Samples

17 patients with febrile UTI agreed to participate in this study. Of these, 10 were included in the study and administered antibiotics according to the guidelines published by the Japanese Association for Infectious Diseases/the Japanese Society of Chemotherapy; these patients did not require dose reduction due to renal dysfunction. On the other hand, seven patients were not included in the study, as they met the exclusion criteria.

**FIGURE 3:**
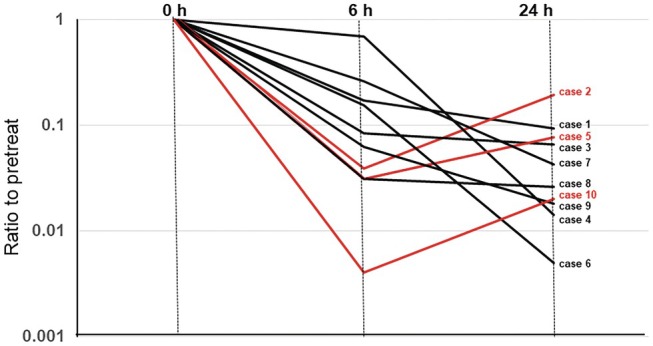
Changes in the number of bacteria (ratio to 0 h) determined by UF‐5000. Black lines indicate the cases with decreased bacterial count at 24 h compared to that at 6 h after empirical antibiotic treatment. Red lines indicate the cases with increased bacterial count at 24 h compared to that at 6 h after empirical treatment.

Gram staining revealed a clear decrease in bacterial counts 6 h after antibiotic therapy in almost all cases in which the causative bacteria were determined to be susceptible to empiric antibiotics by AST. The BACT scattergram confirmed a marked decrease in bacterial counts in all cases (Table 1 and Figure 3).

**TABLE 1:** Clinical and laboratory features of the inpatients and the ratio of bacterial count postantibiotic treatment to pretreatment count using UF‐5000.

No.	Patient information					Urine culture				Blood culture	Bacterial counts by UF‐5000 (BACT)					Clinical outcome
											Pretreat	6 h		24 h		
	Gender	Age	Diagnosis	Underlying disease	Empiric antibiotics	Identified bacteria	Amount (cfu/mL)	Counts	AST S/R	Identified bacteria	Counts (/μL)	Counts (/μL)	Ratio to pretreat	Counts (/μL)	Ratio to pretreat	In 3 days
1	M	86	Pyelonephritis	Ileal conduit	TAZ/PIPC	*Klebsiella pneumoniae*	10^6^	4+	S	ND	6577	1128	0.172	602	0.092	Improvement
						*Escherichia coli*		4+	S							
						*Enterococcus faecalis*		3+	ND							
2	F	76	Pyelonephritis	Hydronephrosis	CTRX	*Morganella morganii*	10^6^	1+	S	ND	150 340	5803	0.039	29 181	0.194	Delayed improvement
						*Klebsiella pneumoniae*		4+	S							
3	M	59	Pyelonephritis	Hydronephrosis	MEPM	*Enterococcus faecalis*	10^6^	1+	ND	ND	181 330	14 964	0.083	11 928	0.066	Improvement
				Ureteral Stent		*Enterococcus avium*		1+	ND							
						*Corynebacterium* sp.		1+	R							
						*Prevotella* sp.		4+	S							
4	M	85	Pyelonephritis	Urethral stricture	MEPM	*Staphylococcus aureus*	10^6^	1+	ND	Negative	161 275	110312	0.684	2240	0.014	Improvement
				Cystostomy		*Proteus mirabilis*		3+	S							
5	F	44	Pyelonephritis	Pyelonephrostomy	CTRX	*Klebsiella pneumoniae*	10^6^	4+	S	ND	50 120	1531	0.031	3800	0.076	Failure
6	M	73	Pyelonephritis	Diabetes	CTRX	*Escherichia coli*	10^6^	4+	S	*Escherichia coli*	49 092	7532	0.153	249	0.005	Improvement
				Urinary retention												
7	M	80	Pyelonephritis	Nephroureterectomy	MEPM	*Escherichia coli* (ESBL)	10^6^	4+	S	ND	22 962	5990	0.261	968	0.042	Improvement
				Urinary catheter		*Citrobacter koseri*		4+	S							
						*Enterococcus faecalis*		4+	ND							
8	F	73	Pyelonephritis	Ureteritis	TAZ/PIPC	*Enterococcus faecalis*	10^6^	2+	ND	ND	2552	80	0.031	67	0.026	Improvement
9	F	84	Pyelonephritis	Ureteral stent	CTRX	*Escherichia coli*	10^6^	4+	S	ND	156 427	9627	0.062	2815	0.018	Improvement
10	M	72	Pyelonephritis	Ileal conduit	MEPM	*Klebsiella pneumoniae*	10^5^	1+	S	ND	93 295	414	0.004	1903	0.020	Improvement
						*Enterococcus faecium*		1+	ND							
						*Staphylococcus haemolyticus*		1+	ND							

Abbreviations: F, female; M, male; TAZ/PIPC, tazobactam/piperacillin; CTRX, ceftoriaxone; MEPM, meropenem; CMZ, cefmetazole; ESBL, extended‐spectrum β‐lactamase; AST, antimicrobial susceptibility testing; S/R, susceptible/resistant; ND, not determined.

As shown in Table 1 and Figure S4, in cases 4 and 10, the urine culture identification showed a mixed infection of gram‐positive and gram‐negative bacteria, and Gram staining showed that only gram‐negative bacteria disappeared and gram‐positive bacteria remained 6 h after antibiotic therapy, which was also confirmed by the BACT scattergram as the cluster pattern specific to gram‐negative bacteria disappeared and the cluster pattern of gram‐positive bacteria remained. Interestingly, in case 9, the urine culture showed a disagreement with Gram staining results; although only *E. coli* was identified by urine culture, Gram staining showed significant amounts of gram‐positive cocci and gram‐negative bacilli. In this instance, the BACT scattergram showed a similar result to Gram staining, a characteristic pattern of both gram‐positive and gram‐negative bacteria [17] before antibiotic administration, and demonstrated results of a few gram‐positive bacteria 24 h after antibiotic administration. In case 5, filamentation of the bacterial cells was clearly observed via Gram staining 6 h after antibiotic administration. This morphological change was also observed as clusters on the CAST scattergram (circled), consistent with the results from the in vitro culture system. In terms of the AST, in case 8, the susceptibility of identified *E. faecalis* to empirically administrated TAZ/PIPC was not determined; however, Gram staining and UF‐5000 showed significant decrease of bacteria after empiric administration.

Gram staining showed that in most cases, no or few bacteria were detected 24 h after antibiotic administration; these results were congruent with those of the BACT scattergram (Figure S4). However, in three patients (Figure S4: Cases 2, 5, and 10), the BACT scattergram showed a subsequent increase in the bacterial count 24 h after administration (Figure 3: Cases 2, 5, and 10). As shown in the Table, in two of these patients (cases 2 and 5), gram‐negative bacilli were identified and determined to be susceptible to empiric antibiotics by AST; however, the effects of antimicrobial treatment were determined to be inadequate by clinical assessment at 5 and 3 days after antibiotic therapy, respectively. In case 2, the antimicrobial agent was not changed, but long‐term administration for 2 weeks was required, and in case 5, the primary antibiotics (CTRX) were changed to Meropenem. In one patient (case 10), where *K. pneumoniae* was identified and determined to be susceptible to empiric antibiotics by AST, the therapeutic course was identified as favorable 3 days after antibiotic therapy, but febrile UTI recurred 3 days after the discontinuation of antibiotic therapy, and the patient had to undergo multiple antibiotic treatments.

## 4 Discussion

This study evaluated whether measuring urine samples using the UF‐5000 can rapidly predict the efficacy of empiric antibiotics in clinical practice by collecting urine over time from patients with febrile UTI.

In in vitro experiment, both antibiotic‐susceptible and resistant bacilli demonstrated morphological changes such as filamentation [16] after approximately 3 h because of β‐lactam antibiotics effect. When processing bacterial dynamics via UF‐5000, it is important to note that a decrease in bacterial population on the BACT scattergram does not directly reflect the decrease in the actual bacterial population. However, upon further agglutination and lysis of antibiotic‐susceptible bacilli, a marked decrease in the number of bacteria was observed 6 h after culturing with antibiotics. Notably, the results of antibiotic‐resistant bacilli suggested that it was not appropriate to determine the efficacy of antibiotics by the bacterial count on the BACT scattergram at 3 h after addition and that the most optimal condition is to monitor the bacterial count on the BACT scattergram at 6 and 24 h after the addition of antibiotics. When fluoroquinolones: Levofloxacin (LVFX) were added to a clinical isolate *E. coli* at intermediate concentrations, the bacteria did not show remarkable filamentation and retained their bacterial shape even after 6 h, suggesting that the optimal timing using UF‐5000 to monitor the decrease of the bacterial population caused by fluoroquinolones should be determined separately from the case treated with β‐lactam antibiotics (data not shown). In terms of gram‐positive cocci, TAZ/PIPC‐susceptible *E. faecalis* showed a substantial decrease in bacterial counts 6 h after culturing with TAZ/PIPC, earlier than LVFX‐susceptible *E. faecalis* , which was observed 24 h after culturing with LVFX (data not shown). It was considered that fluoroquinolone and β‐lactam antibiotics may have different effects on morphological changes of bacterial cells [18, 19]. Our findings suggest that β‐lactam antibiotic treatment can be assessed earlier than fluoroquinolone treatment.

In clinical cases, filamentation was also observed by Gram staining, and this form of bacteria was confirmed by the CAST scattergram (Figure S4: cases 5), suggesting that the phenomena observed in the in vitro system can be reproduced in clinical practice. In one case, even though only gram‐negative bacilli were identified by bacterial culture before antibiotic treatment, gram‐positive and gram‐negative bacteria were confirmed by both Gram staining and BACT scattergram (Figure S4: cases 9) [17], suggesting that UF‐5000 could be an alternative method to Gram staining, even in urine samples containing fastidious bacteria [7, 20].

As empiric antibiotics generally showed good susceptibility to the dominant pathogen, there were no resistant cases; in this study, UF‐5000 showed a remarkable decrease in bacterial counts 6 h after empirical antibiotic administration. Interestingly, in 7 of the 10 cases, the UF‐5000 showed that the bacterial count continued to decrease further at 24 h compared to that at 6 h after antimicrobial treatment, and the clinical manifestations on day 3 also improved. In the other three cases, bacterial counts by UF‐5000, not by Gram staining, increased again 24 h after antibiotic administration (Figure 3), and clinical manifestations on day 3 showed no improvement or recurrence. These results suggest that the key predictive factor for early evaluation was the change in BACT counts from 6 to 24 h.

A revision of the strategy during antibiotic therapy is required in some patients. It is assumed that even if the causative organism is susceptible to the antibiotics administered empirically, the patient's immune status [21, 22], presence of coexisting urinary tract or systemic complications, and severity of sepsis [23] may not always lead to the desired course of complicated febrile UTIs. Because most of the patients included in this clinical study had complicated elements, they possibly showed a tendency for the AST results to not always be consistent with the clinical course. Although this study alone does not provide sufficient evidence to determine whether the susceptibility of empiric antibiotics can be assessed using UF‐5000 without waiting for AST results, our findings suggest that serial urine measurements with UF‐5000 may help predict treatment failure—even when the causative organisms are susceptible to the empirically administered antibiotics.

This study has some limitations. First, the sample size is small; however, as a preliminary study, it appears to have yielded clinically meaningful results. To gain a more comprehensive set of outcomes, we plan to evaluate bacteriuria monitoring using UF‐5000 in the context of multicenter studies. In addition, we aim to expand our research to other cases, including prostatitis, emphysematous pyelonephritis, and other severe infections. Second, we did not show the clinical course and results, as measured by UF‐5000, when the causative organisms were resistant to the antibiotics administered empirically. Third, because most of the cases included in this study contained complicated UTIs, it is still necessary to conduct similar studies in uncomplicated UTIs, although we could guess that such studies would yield clearer results when they are limited to patients with less severe uncomplicated UTIs. By accumulating and analyzing the results of more cases using machine learning, it may be possible to build an algorithm to predict treatment outcomes.

## Author Contributions

**Motohiro Taguchi:** investigation, writing – original draft, writing – review and editing. **Masako Kaido:** conceptualization, writing – original draft, writing – review and editing, data curation, investigation. **Naoki Toya:** conceptualization, writing – review and editing. **Kumiko Yamada:** data curation. **Akihiro Kanematsu:** project administration. **Toeki Yanagi:** data curation. **Shingo Yamamoto:** supervision, writing – original draft, writing – review and editing, funding acquisition.

## Ethics Statement

Approval of Research Protocol by Institutional Review Board: Hyogo Medical University 202 010–030 and Sysmex Corporation 2020–216 have been approved.

## Consent

Written informed consent was obtained from all patients before the commencement of the experiments.

## Conflicts of Interest

Shingo Yamamoto is an Editorial Board member of the International Journal of Urology and a co‐author of this article. Masako Kaido and Naoki Toya are employees of the Sysmex Corporation.

## Supporting information


**Figure S1.** Exemplary detection in UF‐5000 BACT scattergram, CAST scattergram, and Gram staining from in vitro culture experiments.
(Upper panels) On the BACT scattergram of UF‐5000, bacteria were represented by purple dots, and cell fragments were represented by gray dots. Values shown in the scattergrams were bacterial counts per microliter of culture medium, measured using a UF‐5000.
(Middle panels) On the CAST scattergram of the UF‐5000, cells appeared in the gray dotted clusters, which included normal or small‐sized epithelial cells, budding yeast‐like cells, bacterial chains, and WBC clumps; it was likely to include larger sized structures with weak staining intensity.
(Lower panels) Gram staining; gram‐negative bacteria were stained red.
(S1a, b) *Klebsiella pneumoniae* KP12, a CTRX‐susceptible strain.

**Figure S2.** (S2a, b) *Klebsiella pneumoniae* KP27, a CTRX‐resistant strain.

**Figure S3.** (S3a, b) *Escherichia coli* EC352, a TAZ/PIPC‐resistant strain.

**Figure S4.** Detection in UF‐5000 BACT scattergram, CAST scattergram, and Gram staining of clinical urine samples at each collection point.
(Left, upper panels) On the BACT scattergram of UF‐5000, bacteria are represented by purple dots with their cluster locations varying depending on the types of bacteria, and cell fragments were represented by gray dots. Values shown in the scattergram were bacterial counts per microliter of urine, measured using a UF‐5000.
(Left, lower panels) On the CAST scattergram of UF‐5000, cells appeared in the gray dotted area, which includes small‐sized epithelial cells, budding yeast‐like cells, bacterial chains, and WBC clumps; it was likely to include larger sized structures with weak staining intensity.
(Right panels) Gram staining; gram‐negative bacteria were stained red and gram‐positive bacteria were stained blue; semiquantitative bacterial count determined using Gram staining observations. *: Microscopic observation of gram‐stained bacterial cells was performed, but no imaging was available.
**Acronyms**: GPC, gram‐positive cocci; GPR, gram‐positive rods; GNR, gram‐negative rods.

**Data S1.** Figure S4 Continued.
