# Supplementary material for: Clinical Usefulness of Early Evaluation of the Bacteriological Effect of Antibiotics Administered as Empiric Therapy Using the Fully Automated Urine Particle Analyzer UF‐5000 in Febrile Urinary Tract Infections
Source: Int J Urol. 2025 Aug 10;32(11):1604–13. doi: 10.1111/iju.70190 (PMC12586784; doi:10.1111/iju.70190)

Figure S1a

CTRX: 0 µg/mL, *Klebsiella pneumoniae* KP12

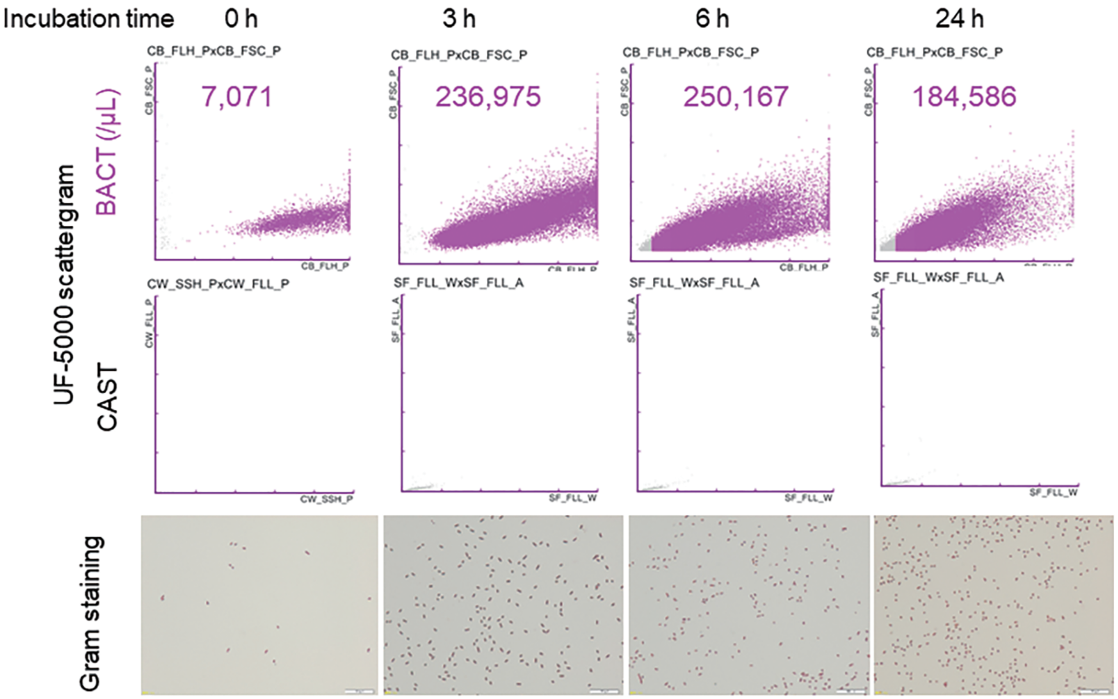

Figure S1b

CTRX: 2 µg/mL, *Klebsiella pneumoniae* KP12

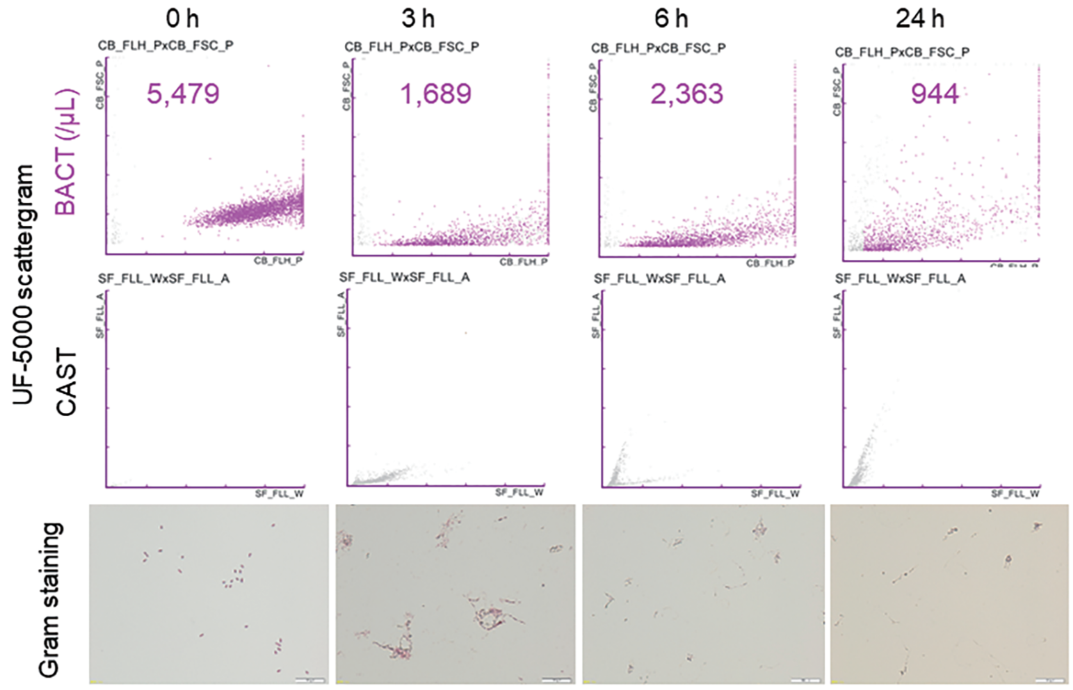

Supplement: Supplementary file 1 — Figure S1. Exemplary detection in UF‐5000 BACT scattergram, CAST scattergram, and Gram staining from in vitro culture experiments. (Upper panels) On the BACT scattergram of UF‐5000, bacteria were represented by purple dots, and cell fragments were represented by gray dots. Values shown in the scattergrams were bacterial counts per microliter of culture medium, measured using a UF‐5000. (Middle panels) On the CAST scattergram of the UF‐5000, cells appeared in the gray dotted clusters, which included normal or small‐sized epithelial cells, budding yeast‐like cells, bacterial chains, and WBC clumps; it was likely to include larger sized structures with weak staining intensity. (Lower panels) Gram staining; gram‐negative bacteria were stained red. (S1a, b) Klebsiella pneumoniae KP12, a CTRX‐susceptible strain. [file IJU-32-1604-s003.pdf]
