# Supplementary material for: Clinical Usefulness of Early Evaluation of the Bacteriological Effect of Antibiotics Administered as Empiric Therapy Using the Fully Automated Urine Particle Analyzer UF‐5000 in Febrile Urinary Tract Infections
Source: Int J Urol. 2025 Aug 10;32(11):1604–13. doi: 10.1111/iju.70190 (PMC12586784; doi:10.1111/iju.70190)

Figure S2a

CTRX: 0 μg/mL, *Klebsiella pneumoniae* KP27

Incubation time 0 h 3 h 6 h 24 h

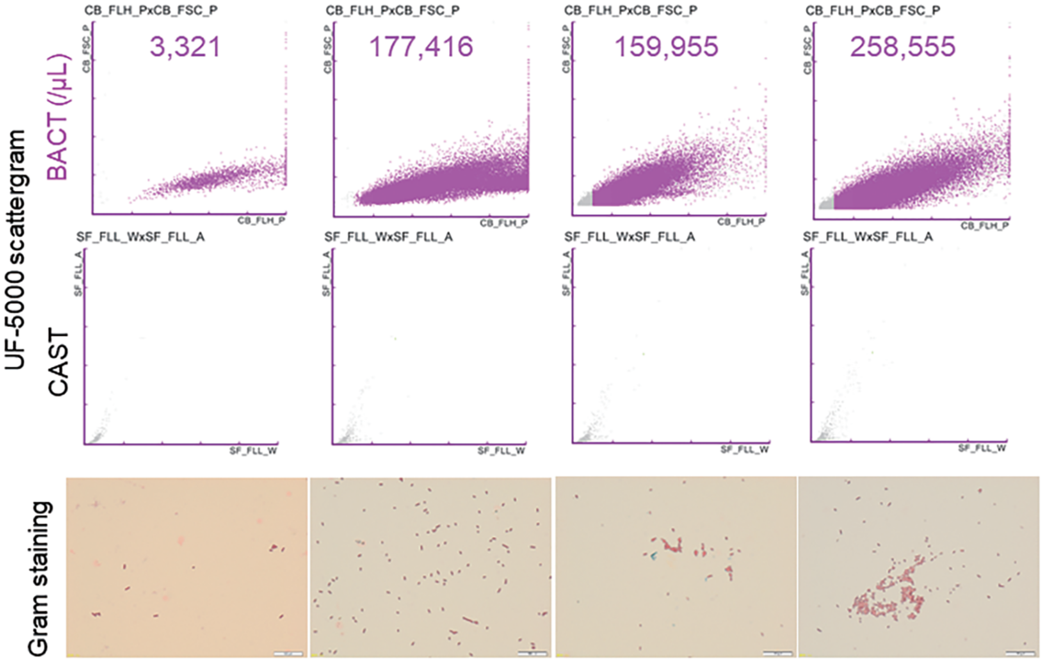

Figure S2b

CTRX: 2 μg/mL, *Klebsiella pneumoniae* KP27

0 h 3 h 6 h 24 h

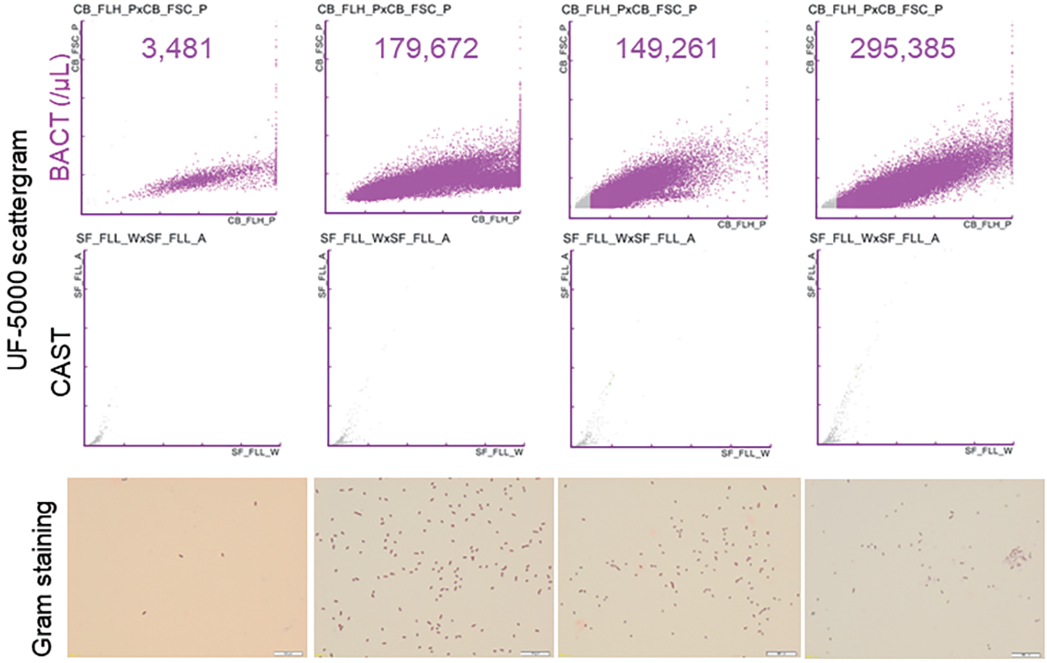

Supplement: Supplementary file 2 — Figure S2. (S2a, b) Klebsiella pneumoniae KP27, a CTRX‐resistant strain. [file IJU-32-1604-s005.pdf]
