# Supplementary material for: Clinical Usefulness of Early Evaluation of the Bacteriological Effect of Antibiotics Administered as Empiric Therapy Using the Fully Automated Urine Particle Analyzer UF‐5000 in Febrile Urinary Tract Infections
Source: Int J Urol. 2025 Aug 10;32(11):1604–13. doi: 10.1111/iju.70190 (PMC12586784; doi:10.1111/iju.70190)

Figure S3a

TAZ/PIPC (1:4) : 0 µg/mL, *Escherichia coli* EC352

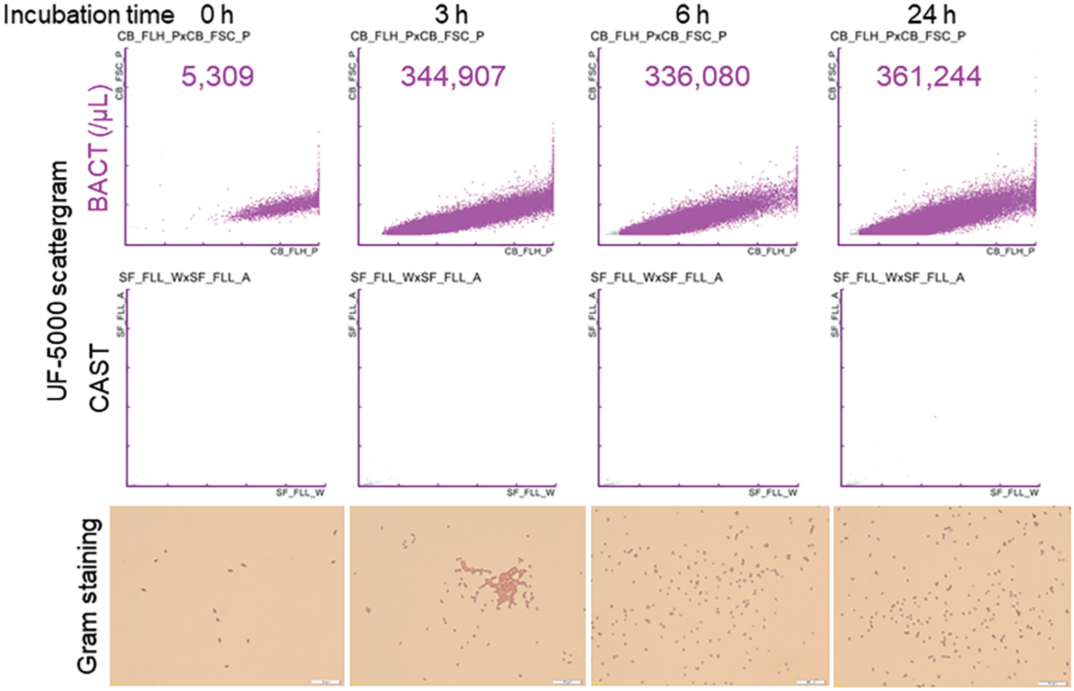

Figure S3b

TAZ/PIPC (1:4) : 32 µg/mL, *Escherichia coli* EC352

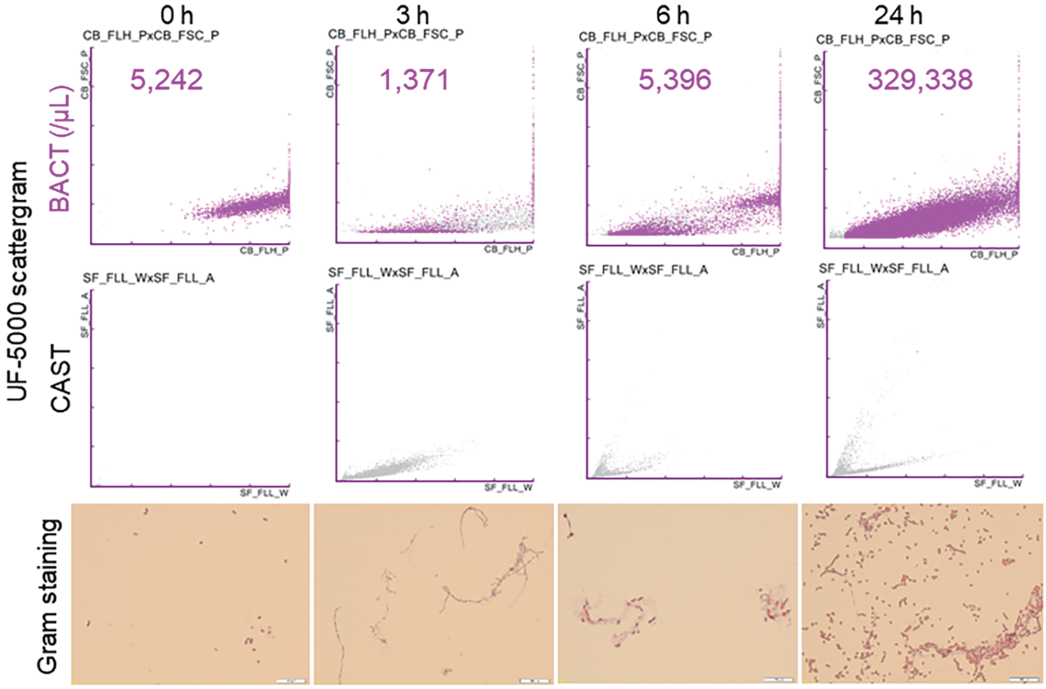

Supplement: Supplementary file 3 — Figure S3. (S3a, b) Escherichia coli EC352, a TAZ/PIPC‐resistant strain. [file IJU-32-1604-s004.pdf]
