# Supplementary figures and images for: Clinical Usefulness of Early Evaluation of the Bacteriological Effect of Antibiotics Administered as Empiric Therapy Using the Fully Automated Urine Particle Analyzer UF‐5000 in Febrile Urinary Tract Infections
Source: Int J Urol. 2025 Aug 10;32(11):1604–13. doi: 10.1111/iju.70190 (PMC12586784; doi:10.1111/iju.70190)

Figure S4

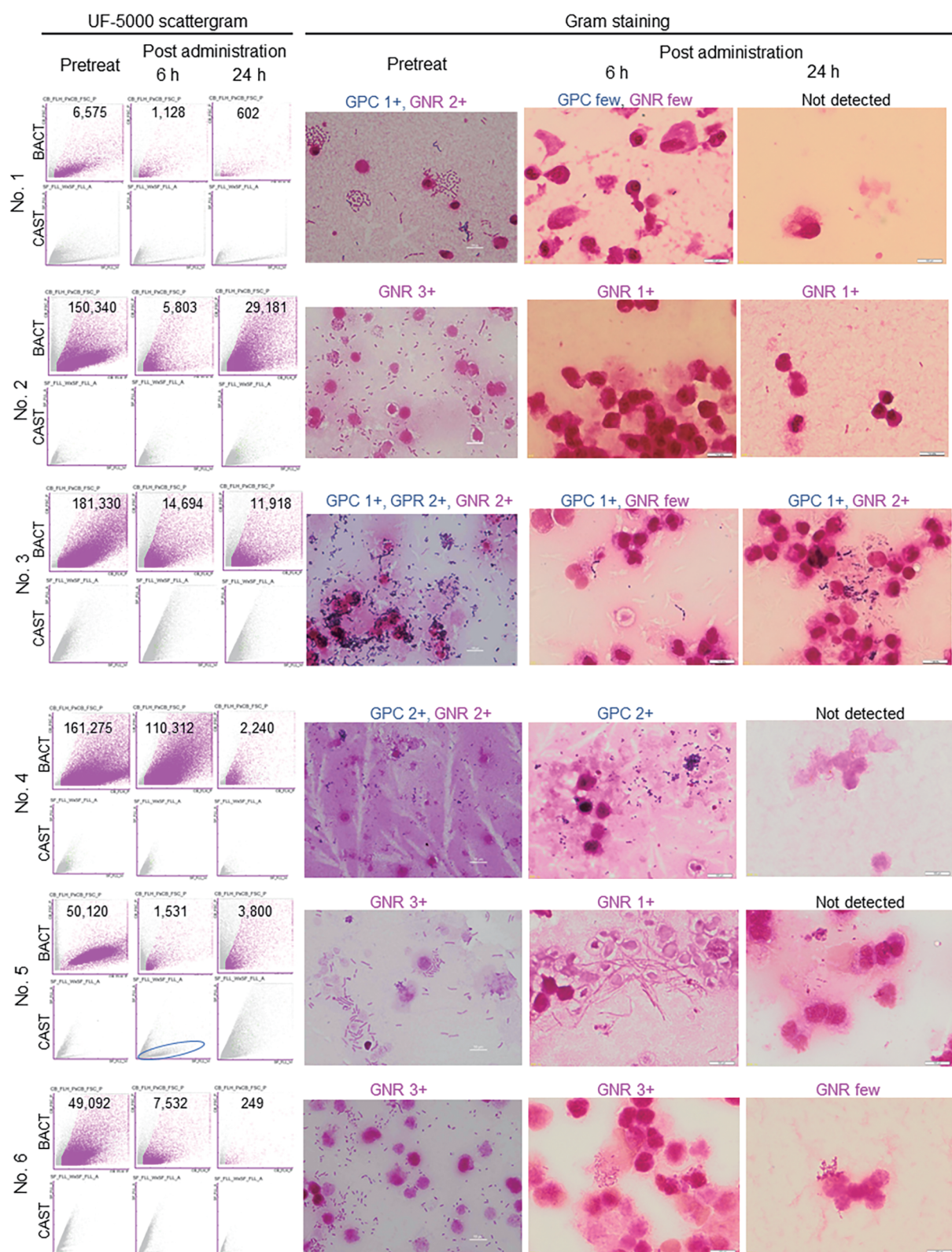

Supplement: Supplementary file 4 — Figure S4. Detection in UF‐5000 BACT scattergram, CAST scattergram, and Gram staining of clinical urine samples at each collection point. (Left, upper panels) On the BACT scattergram of UF‐5000, bacteria are represented by purple dots with their cluster locations varying depending on the types of bacteria, and cell fragments were represented by gray dots. Values shown in the scattergram were bacterial counts per microliter of urine, measured using a UF‐5000. (Left, lower panels) On the CAST scattergram of UF‐5000, cells appeared in the gray dotted area, which includes small‐sized epithelial cells, budding yeast‐like cells, bacterial chains, and WBC clumps; it was likely to include larger sized structures with weak staining intensity. (Right panels) Gram staining; gram‐negative bacteria were stained red and gram‐positive bacteria were stained blue; semiquantitative bacterial count determined using Gram staining observations. *: Microscopic observation of gram‐stained bacterial cells was performed, but no imaging was available. Acronyms: GPC, gram‐positive cocci; GPR, gram‐positive rods; GNR, gram‐negative rods. [file IJU-32-1604-s002.pdf]

Figure S4 (continued)

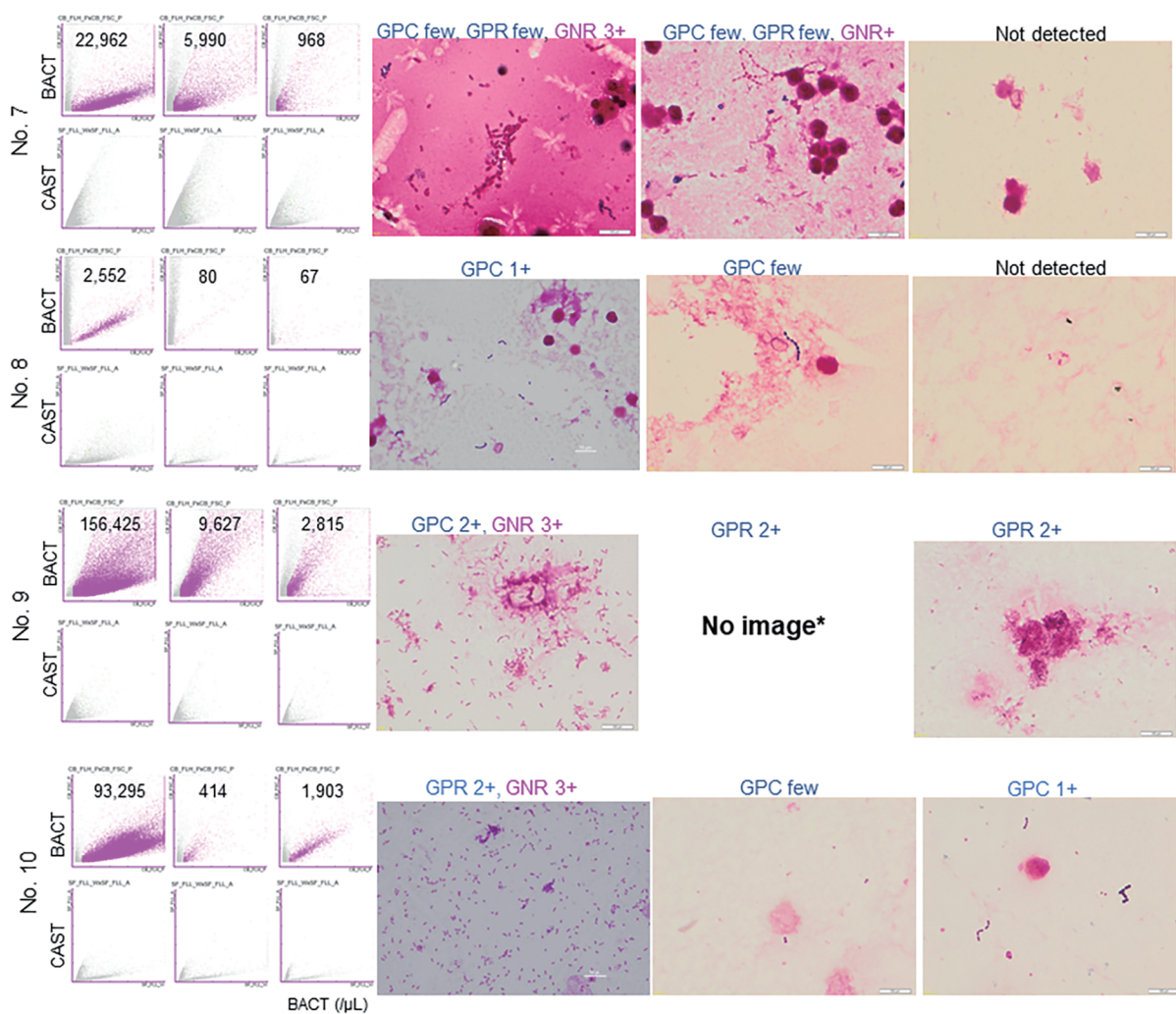

Supplement: Supplementary file 5 — Data S1. Figure S4 Continued. [file IJU-32-1604-s001.pdf]
